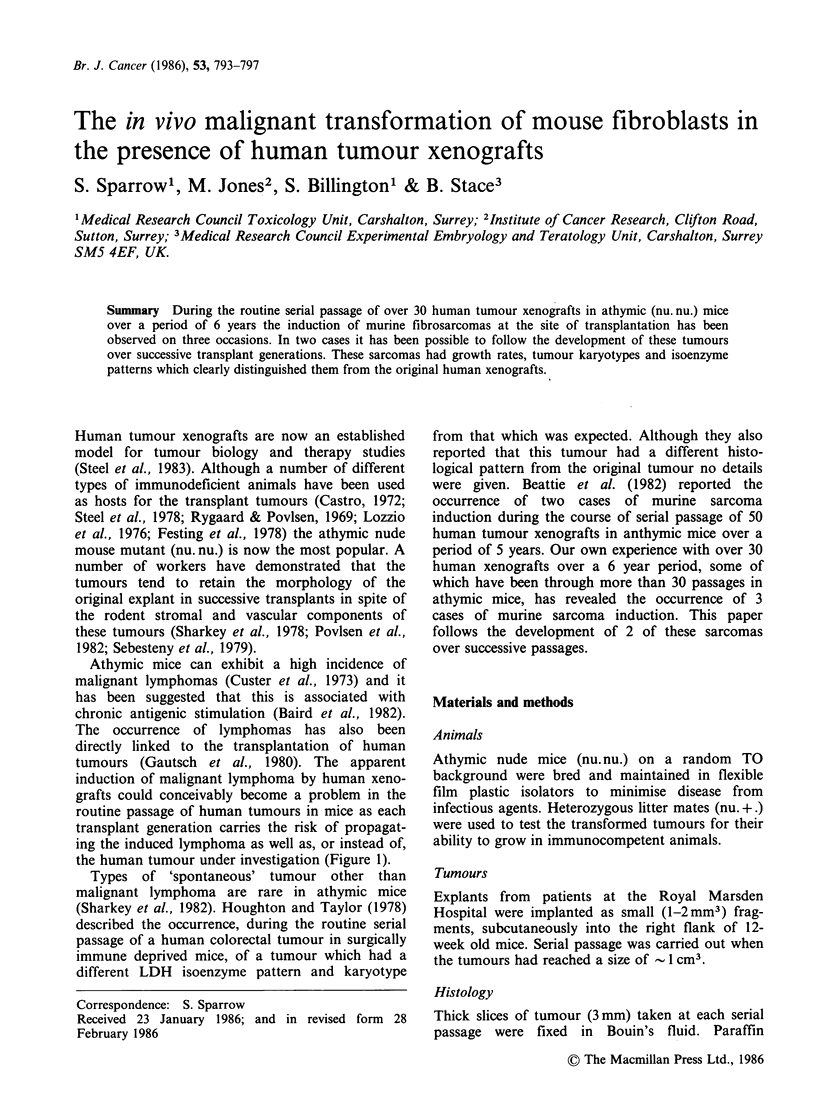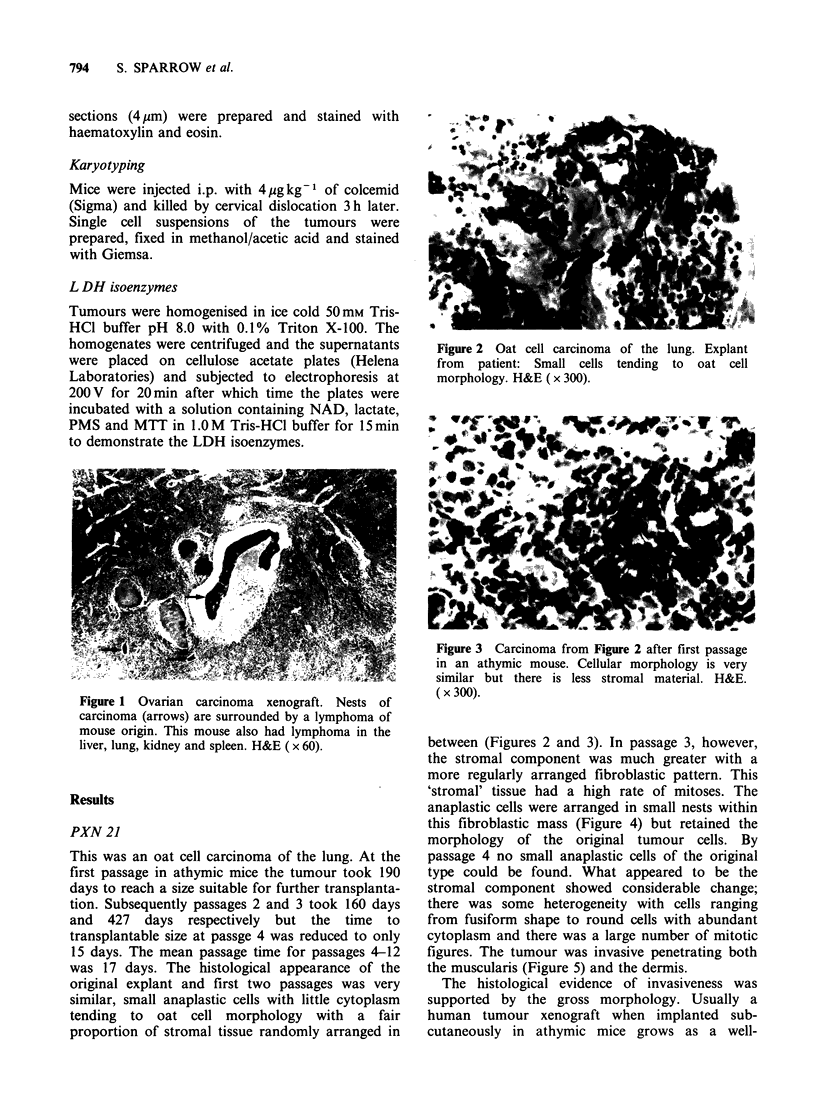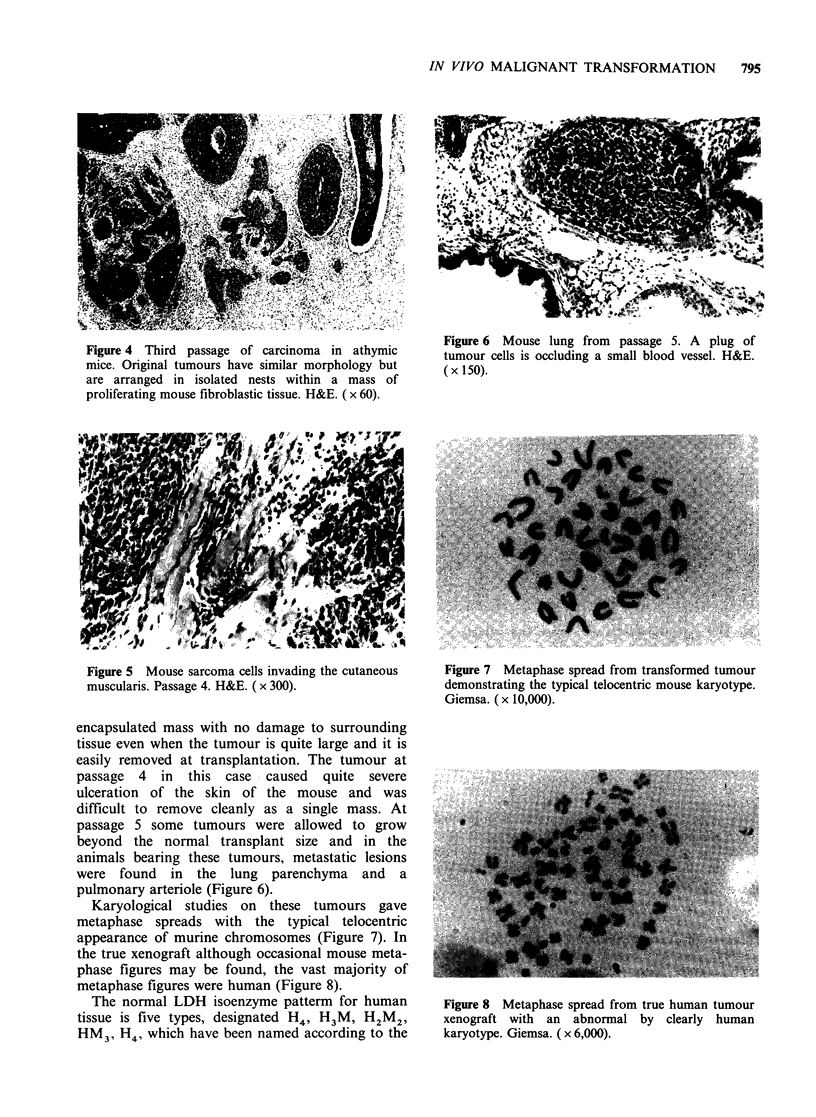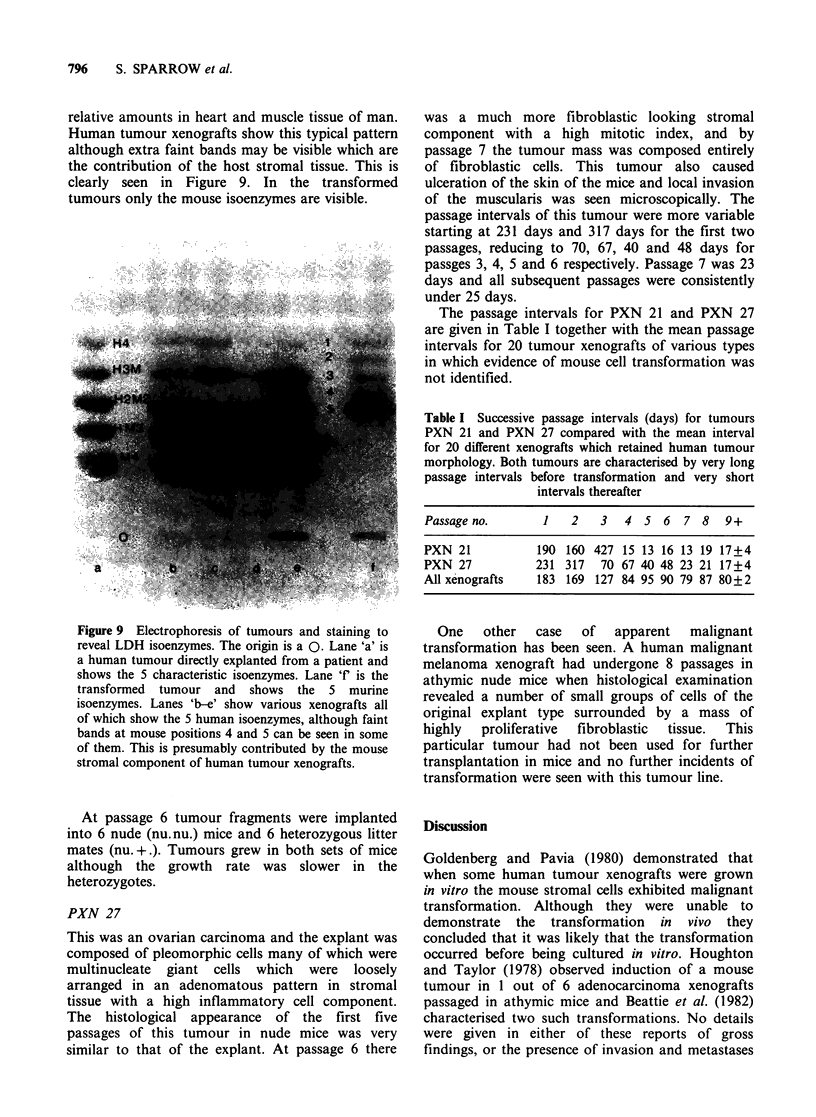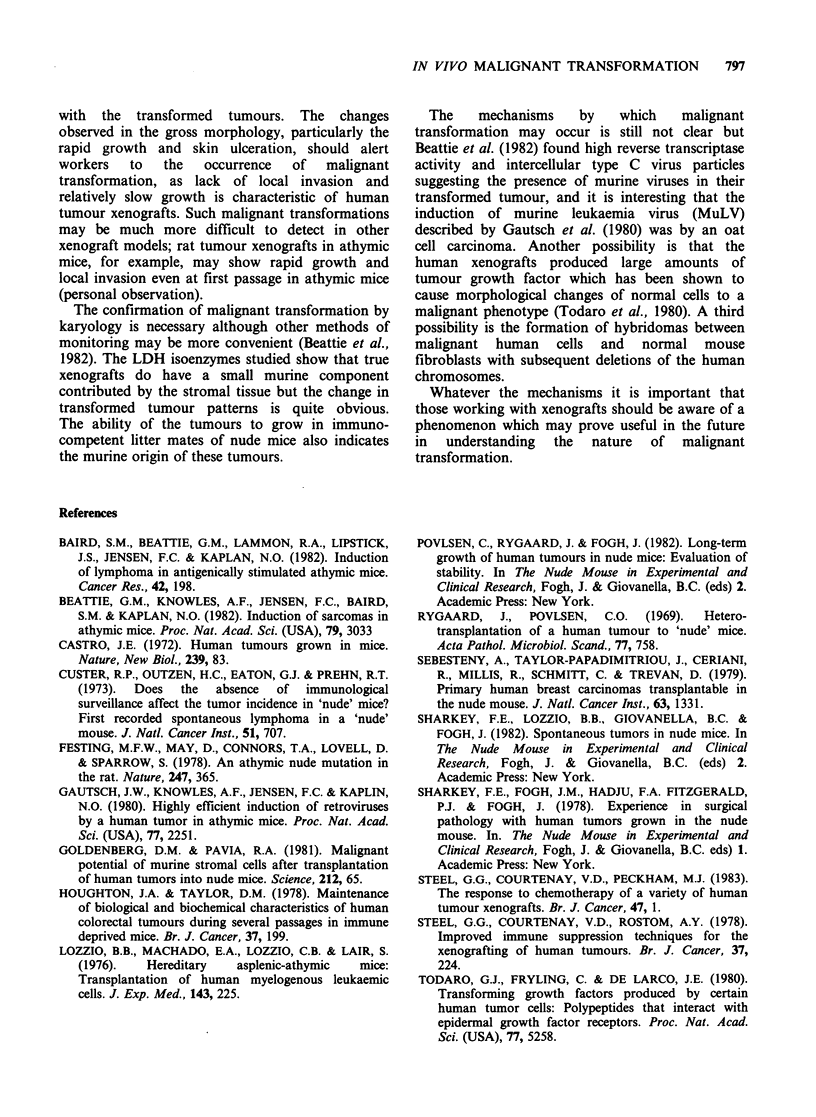# The in vivo malignant transformation of mouse fibroblasts in the presence of human tumour xenografts.

**DOI:** 10.1038/bjc.1986.134

**Published:** 1986-06

**Authors:** S. Sparrow, M. Jones, S. Billington, B. Stace

## Abstract

**Images:**


Br. J. Cancer (1986), 53, 793–797

### The *in vivo* malignant transformation of mouse fibroblasts in the presence of human tumour xenografts

S. Sparrow[1], M. Jones[2], S. Billington[1] & B. Stace[3]

[1]*Medical Research Council Toxicology Unit, Carshalton, Surrey;* [2]*Institute of Cancer Research, Clifton Road, Sutton, Surrey;* [3]*Medical Research Council Experimental Embryology and Teratology Unit, Carshalton, Surrey SM5 4EF, UK.*

**Summary** During the routine serial passage of over 30 human tumour xenografts in athymic (nu. nu.) mice over a period of 6 years the induction of murine fibrosarcomas at the site of transplantation has been observed on three occasions. In two cases it has been possible to follow the development of these tumours over successive transplant generations. These sarcomas had growth rates, tumour karyotypes and isoenzyme patterns which clearly distinguished them from the original human xenografts.

Human tumour xenografts are now an established model for tumour biology and therapy studies (Steel *et al.*, 1983). Although a number of different types of immunodeficient animals have been used as hosts for the transplant tumours (Castro, 1972; Steel *et al.*, 1978; Rygaard & Povlsen, 1969; Lozzio *et al.*, 1976; Festing *et al.*, 1978) the athymic nude mouse mutant (nu. nu.) is now the most popular. A number of workers have demonstrated that the tumours tend to retain the morphology of the original explant in successive transplants in spite of the rodent stromal and vascular components of these tumours (Sharkey *et al.*, 1978; Povlsen *et al.*, 1982; Sebesteny *et al.*, 1979).

Athymic mice can exhibit a high incidence of malignant lymphomas (Custer *et al.*, 1973) and it has been suggested that this is associated with chronic antigenic stimulation (Baird *et al.*, 1982). The occurrence of lymphomas has also been directly linked to the transplantation of human tumours (Gautsch *et al.*, 1980). The apparent induction of malignant lymphoma by human xenografts could conceivably become a problem in the routine passage of human tumours in mice as each transplant generation carries the risk of propagating the induced lymphoma as well as, or instead of, the human tumour under investigation (Figure 1).

Types of 'spontaneous' tumour other than malignant lymphoma are rare in athymic mice (Sharkey *et al.*, 1982). Houghton and Taylor (1978) described the occurrence, during the routine serial passage of a human colorectal tumour in surgically immune deprived mice, of a tumour which had a different LDH isoenzyme pattern and karyotype from that which was expected. Although they also reported that this tumour had a different histological pattern from the original tumour no details were given. Beattie *et al.* (1982) reported the occurrence of two cases of murine sarcoma induction during the course of serial passage of 50 human tumour xenografts in anthymic mice over a period of 5 years. Our own experience with over 30 human xenografts over a 6 year period, some of which have been through more than 30 passages in athymic mice, has revealed the occurrence of 3 cases of murine sarcoma induction. This paper follows the development of 2 of these sarcomas over successive passages.

### Materials and methods

### *Animals*

Athymic nude mice (nu.nu.) on a random TO background were bred and maintained in flexible film plastic isolators to minimise disease from infectious agents. Heterozygous litter mates (nu. +.) were used to test the transformed tumours for their ability to grow in immunocompetent animals.

### *Tumours*

Explants from patients at the Royal Marsden Hospital were implanted as small (1–2 mm$^3$) fragments, subcutaneously into the right flank of 12-week old mice. Serial passage was carried out when the tumours had reached a size of $\sim 1\,cm^3$.

### *Histology*

Thick slices of tumour (3 mm) taken at each serial passage were fixed in Bouin's fluid. Paraffin

Correspondence: S. Sparrow
Received 23 January 1986; and in revised form 28 February 1986

© The Macmillan Press Ltd., 1986

sections (4 $\mu$m) were prepared and stained with haematoxylin and eosin.

### *Karyotyping*

Mice were injected i.p. with 4 $\mu g\,kg^{-1}$ of colcemid (Sigma) and killed by cervical dislocation 3 h later. Single cell suspensions of the tumours were prepared, fixed in methanol/acetic acid and stained with Giemsa.

### *L DH isoenzymes*

Tumours were homogenised in ice cold 50 mM Tris-HCl buffer pH 8.0 with 0.1% Triton X-100. The homogenates were centrifuged and the supernatants were placed on cellulose acetate plates (Helena Laboratories) and subjected to electrophoresis at 200 V for 20 min after which time the plates were incubated with a solution containing NAD, lactate, PMS and MTT in 1.0 M Tris-HCl buffer for 15 min to demonstrate the LDH isoenzymes.

**Figure 1** Ovarian carcinoma xenograft. Nests of carcinoma (arrows) are surrounded by a lymphoma of mouse origin. This mouse also had lymphoma in the liver, lung, kidney and spleen. H&E ( × 60).

### Results

### *PXN 21*

This was an oat cell carcinoma of the lung. At the first passage in athymic mice the tumour took 190 days to reach a size suitable for further transplantation. Subsequently passages 2 and 3 took 160 days and 427 days respectively but the time to transplantable size at passge 4 was reduced to only 15 days. The mean passage time for passages 4–12 was 17 days. The histological appearance of the original explant and first two passages was very similar, small anaplastic cells with little cytoplasm tending to oat cell morphology with a fair proportion of stromal tissue randomly arranged in between (Figures 2 and 3). In passage 3, however, the stromal component was much greater with a more regularly arranged fibroblastic pattern. This 'stromal' tissue had a high rate of mitoses. The anaplastic cells were arranged in small nests within this fibroblastic mass (Figure 4) but retained the morphology of the original tumour cells. By passage 4 no small anaplastic cells of the original type could be found. What appeared to be the stromal component showed considerable change; there was some heterogeneity with cells ranging from fusiform shape to round cells with abundant cytoplasm and there was a large number of mitotic figures. The tumour was invasive penetrating both the muscularis (Figure 5) and the dermis.

**Figure 2** Oat cell carcinoma of the lung. Explant from patient: Small cells tending to oat cell morphology. H&E ( × 300).

**Figure 3** Carcinoma from **Figure 2** after first passage in an athymic mouse. Cellular morphology is very similar but there is less stromal material. H&E. ( × 300).

The histological evidence of invasiveness was supported by the gross morphology. Usually a human tumour xenograft when implanted subcutaneously in athymic mice grows as a well-

**Figure 4** Third passage of carcinoma in athymic mice. Original tumours have similar morphology but are arranged in isolated nests within a mass of proliferating mouse fibroblastic tissue. H&E. ( × 60).

**Figure 6** Mouse lung from passage 5. A plug of tumour cells is occluding a small blood vessel. H&E. ( × 150).

**Figure 5** Mouse sarcoma cells invading the cutaneous muscularis. Passage 4. H&E. ( × 300).

**Figure 7** Metaphase spread from transformed tumour demonstrating the typical telocentric mouse karyotype. Giemsa. ( × 10,000).

encapsulated mass with no damage to surrounding tissue even when the tumour is quite large and it is easily removed at transplantation. The tumour at passage 4 in this case caused quite severe ulceration of the skin of the mouse and was difficult to remove cleanly as a single mass. At passage 5 some tumours were allowed to grow beyond the normal transplant size and in the animals bearing these tumours, metastatic lesions were found in the lung parenchyma and a pulmonary arteriole (Figure 6).

Karyological studies on these tumours gave metaphase spreads with the typical telocentric appearance of murine chromosomes (Figure 7). In the true xenograft although occasional mouse metaphase figures may be found, the vast majority of metaphase figures were human (Figure 8).

The normal LDH isoenzyme patterm for human tissue is five types, designated $H_4$, $H_3M$, $H_2M_2$, $HM_3$, $H_4$, which have been named according to the

**Figure 8** Metaphase spread from true human tumour xenograft with an abnormal by clearly human karyotype. Giemsa. ( × 6,000).

relative amounts in heart and muscle tissue of man. Human tumour xenografts show this typical pattern although extra faint bands may be visible which are the contribution of the host stromal tissue. This is clearly seen in Figure 9. In the transformed tumours only the mouse isoenzymes are visible.

**Figure 9** Electrophoresis of tumours and staining to reveal LDH isoenzymes. The origin is a O. Lane 'a' is a human tumour directly explanted from a patient and shows the 5 characteristic isoenzymes. Lane 'f' is the transformed tumour and shows the 5 murine isoenzymes. Lanes 'b–e' show various xenografts all of which show the 5 human isoenzymes, although faint bands at mouse positions 4 and 5 can be seen in some of them. This is presumably contributed by the mouse stromal component of human tumour xenografts.

At passage 6 tumour fragments were implanted into 6 nude (nu.nu.) mice and 6 heterozygous litter mates (nu.+.). Tumours grew in both sets of mice although the growth rate was slower in the heterozygotes.

*PXN 27*

This was an ovarian carcinoma and the explant was composed of pleomorphic cells many of which were multinucleate giant cells which were loosely arranged in an adenomatous pattern in stromal tissue with a high inflammatory cell component. The histological appearance of the first five passages of this tumour in nude mice was very similar to that of the explant. At passage 6 there was a much more fibroblastic looking stromal component with a high mitotic index, and by passage 7 the tumour mass was composed entirely of fibroblastic cells. This tumour also caused ulceration of the skin of the mice and local invasion of the muscularis was seen microscopically. The passage intervals of this tumour were more variable starting at 231 days and 317 days for the first two passages, reducing to 70, 67, 40 and 48 days for passges 3, 4, 5 and 6 respectively. Passage 7 was 23 days and all subsequent passages were consistently under 25 days.

The passage intervals for PXN 21 and PXN 27 are given in Table I together with the mean passage intervals for 20 tumour xenografts of various types in which evidence of mouse cell transformation was not identified.

**Table I** Successive passage intervals (days) for tumours PXN 21 and PXN 27 compared with the mean interval for 20 different xenografts which retained human tumour morphology. Both tumours are characterised by very long passage intervals before transformation and very short intervals thereafter

| *Passage no.* | *1* | *2* | *3* | *4* | *5* | *6* | *7* | *8* | *9+* |
|---|---|---|---|---|---|---|---|---|---|
| PXN 21 | 190 | 160 | 427 | 15 | 13 | 16 | 13 | 19 | 17±4 |
| PXN 27 | 231 | 317 | 70 | 67 | 40 | 48 | 23 | 21 | 17±4 |
| All xenografts | 183 | 169 | 127 | 84 | 95 | 90 | 79 | 87 | 80±2 |

One other case of apparent malignant transformation has been seen. A human malignant melanoma xenograft had undergone 8 passages in athymic nude mice when histological examination revealed a number of small groups of cells of the original explant type surrounded by a mass of highly proliferative fibroblastic tissue. This particular tumour had not been used for further transplantation in mice and no further incidents of transformation were seen with this tumour line.

### Discussion

Goldenberg and Pavia (1980) demonstrated that when some human tumour xenografts were grown *in vitro* the mouse stromal cells exhibited malignant transformation. Although they were unable to demonstrate the transformation *in vivo* they concluded that it was likely that the transformation occurred before being cultured *in vitro*. Houghton and Taylor (1978) observed induction of a mouse tumour in 1 out of 6 adenocarcinoma xenografts passaged in athymic mice and Beattie *et al.* (1982) characterised two such transformations. No details were given in either of these reports of gross findings, or the presence of invasion and metastases with the transformed tumours. The changes observed in the gross morphology, particularly the rapid growth and skin ulceration, should alert workers to the occurrence of malignant transformation, as lack of local invasion and relatively slow growth is characteristic of human tumour xenografts. Such malignant transformations may be much more difficult to detect in other xenograft models; rat tumour xenografts in athymic mice, for example, may show rapid growth and local invasion even at first passage in athymic mice (personal observation).

The confirmation of malignant transformation by karyology is necessary although other methods of monitoring may be more convenient (Beattie *et al.*, 1982). The LDH isoenzymes studied show that true xenografts do have a small murine component contributed by the stromal tissue but the change in transformed tumour patterns is quite obvious. The ability of the tumours to grow in immunocompetent litter mates of nude mice also indicates the murine origin of these tumours.

The mechanisms by which malignant transformation may occur is still not clear but Beattie *et al.* (1982) found high reverse transcriptase activity and intercellular type C virus particles suggesting the presence of murine viruses in their transformed tumour, and it is interesting that the induction of murine leukaemia virus (MuLV) described by Gautsch *et al.* (1980) was by an oat cell carcinoma. Another possibility is that the human xenografts produced large amounts of tumour growth factor which has been shown to cause morphological changes of normal cells to a malignant phenotype (Todaro *et al.*, 1980). A third possibility is the formation of hybridomas between malignant human cells and normal mouse fibroblasts with subsequent deletions of the human chromosomes.

Whatever the mechanisms it is important that those working with xenografts should be aware of a phenomenon which may prove useful in the future in understanding the nature of malignant transformation.

**References**

BAIRD, S.M., BEATTIE, G.M., LAMMON, R.A., LIPSTICK, J.S., JENSEN, F.C. & KAPLAN, N.O. (1982). Induction of lymphoma in antigenically stimulated athymic mice. *Cancer Res.*, **42**, 198.

BEATTIE, G.M., KNOWLES, A.F., JENSEN, F.C., BAIRD, S.M. & KAPLAN, N.O. (1982). Induction of sarcomas in athymic mice. *Proc. Nat. Acad. Sci.* (USA), **79**, 3033

CASTRO, J.E. (1972). Human tumours grown in mice. *Nature, New Biol.*, **239**, 83.

CUSTER, R.P., OUTZEN, H.C., EATON, G.J. & PREHN, R.T. (1973). Does the absence of immunological surveillance affect the tumor incidence in 'nude' mice? First recorded spontaneous lymphoma in a 'nude' mouse. *J. Natl. Cancer Inst.*, **51**, 707.

FESTING, M.F.W., MAY, D., CONNORS, T.A., LOVELL, D. & SPARROW, S. (1978). An athymic nude mutation in the rat. *Nature*, **247**, 365.

GAUTSCH, J.W., KNOWLES, A.F., JENSEN, F.C. & KAPLIN, N.O. (1980). Highly efficient induction of retroviruses by a human tumor in athymic mice. *Proc. Nat. Acad. Sci.* (USA), **77**, 2251.

GOLDENBERG, D.M. & PAVIA, R.A. (1981). Malignant potential of murine stromal cells after transplantation of human tumors into nude mice. *Science*, **212**, 65.

HOUGHTON, J.A. & TAYLOR, D.M. (1978). Maintenance of biological and biochemical characteristics of human colorectal tumours during several passages in immune deprived mice. *Br. J. Cancer*, **37**, 199.

LOZZIO, B.B., MACHADO, E.A., LOZZIO, C.B. & LAIR, S. (1976). Hereditary asplenic-athymic mice: Transplantation of human myelogenous leukaemic cells. *J. Exp. Med.*, **143**, 225.

POVLSEN, C., RYGAARD, J. & FOGH, J. (1982). Long-term growth of human tumours in nude mice: Evaluation of stability. In *The Nude Mouse in Experimental and Clinical Research*, Fogh, J. & Giovanella, B.C. (eds) **2**. Academic Press: New York.

RYGAARD, J., POVLSEN, C.O. (1969). Heterotransplantation of a human tumour to 'nude' mice. *Acta Pathol. Microbiol. Scand.*, **77**, 758.

SEBESTENY, A., TAYLOR-PAPADIMITRIOU, J., CERIANI, R., MILLIS, R., SCHMITT, C. & TREVAN, D. (1979). Primary human breast carcinomas transplantable in the nude mouse. *J. Natl. Cancer Inst.*, **63**, 1331.

SHARKEY, F.E., LOZZIO, B.B., GIOVANELLA, B.C. & FOGH, J. (1982). Spontaneous tumors in nude mice. In *The Nude Mouse in Experimental and Clinical Research*, Fogh, J. & Giovanella, B.C. (eds) **2**. Academic Press: New York.

SHARKEY, F.E., FOGH, J.M., HADJU, F.A. FITZGERALD, P.J. & FOGH, J. (1978). Experience in surgical pathology with human tumors grown in the nude mouse. In. *The Nude Mouse in Experimental and Clinical Research*, Fogh, J. & Giovanella, B.C. eds) **1**. Academic Press: New York.

STEEL, G.G., COURTENAY, V.D., PECKHAM, M.J. (1983). The response to chemotherapy of a variety of human tumour xenografts. *Br. J. Cancer*, **47**, 1.

STEEL, G.G., COURTENAY, V.D., ROSTOM, A.Y. (1978). Improved immune suppression techniques for the xenografting of human tumours. *Br. J. Cancer*, **37**, 224.

TODARO, G.J., FRYLING, C. & DE LARCO, J.E. (1980). Transforming growth factors produced by certain human tumor cells: Polypeptides that interact with epidermal growth factor receptors. *Proc. Nat. Acad. Sci.* (USA), **77**, 5258.